# An Overrepresentation of High Frequencies in the Mouse Inferior Colliculus Supports the Processing of Ultrasonic Vocalizations

**DOI:** 10.1371/journal.pone.0133251

**Published:** 2015-08-05

**Authors:** Jose A. Garcia-Lazaro, Kathryn N. Shepard, Jason A. Miranda, Robert C. Liu, Nicholas A. Lesica

**Affiliations:** 1 Ear Institute, University College London, 332 Grays Inn Road, London, WC1X 8EE, United Kingdom; 2 Department of Biology, Emory University, 1510 Clifton Road, Atlanta, Georgia, 30322, United States of America; Utrecht University, NETHERLANDS

## Abstract

Mice are of paramount importance in biomedical research and their vocalizations are a subject of interest for researchers across a wide range of health-related disciplines due to their increasingly important value as a phenotyping tool in models of neural, speech and language disorders. However, the mechanisms underlying the auditory processing of vocalizations in mice are not well understood. The mouse audiogram shows a peak in sensitivity at frequencies between 15-25 kHz, but weaker sensitivity for the higher ultrasonic frequencies at which they typically vocalize. To investigate the auditory processing of vocalizations in mice, we measured evoked potential, single-unit, and multi-unit responses to tones and vocalizations at three different stages along the auditory pathway: the auditory nerve and the cochlear nucleus in the periphery, and the inferior colliculus in the midbrain. Auditory brainstem response measurements suggested stronger responses in the midbrain relative to the periphery for frequencies higher than 32 kHz. This result was confirmed by single- and multi-unit recordings showing that high ultrasonic frequency tones and vocalizations elicited responses from only a small fraction of cells in the periphery, while a much larger fraction of cells responded in the inferior colliculus. These results suggest that the processing of communication calls in mice is supported by a specialization of the auditory system for high frequencies that emerges at central stations of the auditory pathway.

## Introduction

Mice are rapidly becoming an important model for auditory research due to the increased availability of genetic tools for manipulating neural circuits. Moreover, mouse communication has become an important model for elucidating the neurobiology of social behaviour [1–4], and mouse vocalizations are being used by researchers as a phenotyping tool for models of neural disorders like autism [5, 6], fragile X syndrome [7], and speech and language disorders [8]. However, there remains a fundamental gap in our understanding of the auditory processing that supports vocal communication in mice: audiograms recorded for the house mouse [9], as well as neurophysiological studies [10–13], indicate maximal sensitivity to sounds with frequencies between 15 and 25 kHz with weaker sensitivity at high ultrasonic frequencies, yet mouse vocalizations regularly exceed 60 kHz [14, 15].

To take full advantage of mouse communication as a research model, we must first understand how their auditory system processes the high frequency vocalizations that they produce. It has recently been suggested that the processing of vocalizations may be supported by nonlinear distortions created by interactions on the basilar membrane [16]. However, as the majority of calls emitted in social encounters have only a single frequency component rather than multiple components required to create cochlear distortions [4, 15], this is unlikely to be a primary mechanism. Here, we analyze evoked potential, single-unit, and multi-unit activity at three different stages along the auditory pathway to provide evidence that communication in mice is supported by the overrepresentation of high frequencies in the central auditory system, i.e. an overrepresentation of high ultrasonic frequencies that amplifies responses to vocalizations.

## Methods

### Auditory brainstem responses (ABR)

All methods described in this section were approved by the Emory IACUC. Experiments were conducted in an anechoic chamber. ABRs were recorded from 18–20 week-old female CBA/CaJ mice anesthetized with a mix of ketamine and xylazine (100 and 10 mg/kg respectively). Subdermal needles were used as electrodes and the acquired signals (System 3, Tucker-Davis Technologies, TDT, Alachua, FL, USA) were processed using standard methods as described in [17]. Stimuli were sampled at 195 kHz and calibrated using a ¼” Bruel and Kjaer (B&K, Denmark) microphone. Stimuli were presented (BioSig, TDT) free field at 74 dB SPL through an Infinity EMIT tweeter placed 90° to the right side of the animal. We recorded responses to positive-going broadband clicks of 0.5 ms duration presented at a rate of 19 per second and 3-ms tone pips (1.5 rise/fall times) with frequencies of 8, 16, 32, 64 and 80 kHz presented at a rate of 21/second with a total of 500 consecutive repeats presented for each stimulus as described in [18]. Signals were sampled at 25 kHz, bandpass filtered (100–3000 Hz) and amplified by a factor of 200,000. ABR thresholds were initially estimated online by reducing the intensity in 10 dB and then 5 dB steps until the lowest intensity at which the dominant ABR wave was clearly visible [17, 19]. Offline, ABRs were analyzed in a blind fashion across animals to determine final thresholds. For analysis of the tone pip-evoked responses, we defined wave I as the first peak that consistently appeared with low temporal variation upon repeated stimulation (e.g. repeatable average peak within ~0.5 ms on successive sets of trials), a hallmark of ABR peaks [19], and occurred within the expected range of the cochlear travelling wave delays for each frequency (approximately up to 1.6 ms range from 8 to 80 kHz) [20]. Wave V was usually identifiable as the final consistently appearing peak, typically following a large, prominent upward slope, occurring around 5.7 ms after stimulus onset. When this prominent slope wasn’t obvious, the local maximum nearest to 5.7 ms was used. The amplitude of wave V was defined as the difference in voltage between the peak occurring at this point in time, and the nearest antecedent trough.

### Single-unit and multi-unit recordings

The experimental protocols described in this section were approved by the United Kingdom Home Office Inspectorate under project licence 30/2481, in conformity with the 1986 Animals Scientific Procedures Act. Experiments were conducted in a sound-insulated chamber (Industrial Acoustics, Winchester, UK). 12–16 week-old female CBA/Ca mice were anaesthetized with a mix of tribromoethanol and tert-amyl alcohol (Sigma-Aldrich, Gillinham, Dorset, UK) prepared as indicated in [21] or a mix of ketamine and xylazine. Anesthesia was maintained via intraperitoneal infusion of the same mix and the dose was adjusted as required to maintain a stable level of anesthesia as assessed by monitoring ECG, respiratory rate and pedal (paw-pinch) withdrawal reflex. The body core temperature was monitored and maintained constant between 37 – 38°C. The skull was exposed by incision of the scalp and a metallic pin was cemented to it and subsequently coupled to a stainless steel head holder. ABRs to clicks with varying intensity were recorded (typically every hour) to provide an indirect coarse assessment of cochlear integrity and the recording session was terminated by an overdose of sodium pentobarbital (Euthatal: Merial Animal Health, Woking, Surrey, UK) if the sensitivity threshold increased by more than 10 dB.

For single-unit recordings in the auditory nerve (AN), the occipital aspects of the left side of the skull were exposed and a craniotomy was performed at the region of the skull overlying the cerebellum. A semicerebellectomy was performed to expose the left cochlear nucleus (CN) and the semicircular canals. Glass microelectrodes with impedances of 8–12 MΩ, filled with 1M NaCl solution, were inserted through the CN at 200 μm medial and 1200 μm rostral relative to the dark pigmented cells in the semicircular canals. To distinguish AN fibers from CN neurons, we estimated the center frequency (CF) and threshold of each neuron, and recorded responses to repeated presentations (typically 500) of the CF tone at 30 dB above threshold. We then used the criterion described in [13], which uses the coefficient of variation (CV) of interspike intervals (ISI) and the first spike latency (FSL), to isolate the responses of AN fibers. Only cells with CVs ≥ 0.5, FSL ≤ 5 ms and primary-like post-stimulus time histograms (PSTHs) were considered for further analysis (see S1 Fig). Spikes from single-units were isolated from voltage recordings as follows: 1) a bandpass filtering was applied between 500 and 5000 Hz, 2) potential spikes were identified as snippets exceeding a threshold (minimum of 0.7 ms between potential spikes), 3) each snippet was projected into the space defined by the first three principal components of all snippets, 4) clusters of snippets within this space were identified using KlustaKwik (http://klustakwik.sourceforge.net), and 5) the likelihood that each cluster represented a single unit was quantified using isolation distance [22]. Only single-unit clusters with an isolation distance greater than 20 were selected for all subsequent analyses.

For multi-unit recordings in the dorsal cochlear nucleus, the craniotomy described above made the dorsal division of the cochlear nucleus (DCN) easily accessible. Multi-unit responses were recorded using 32-channel silicon electrodes arrays (Neuronexus Technologies, Ann Arbor, MI, USA) arranged in 4 shanks with 8 recording sites per shank. In an attempt to sample a large fraction of the DCN across three dimensions in each animal, we performed multiple electrode penetrations. Individual penetrations performed in series (typically 4 in each animal) were spaced at 100 μm intervals along the rostral-caudal axis and oriented along the medial-lateral axis such that each penetration covered an entire plane of the DCN, with most recording sites contained within its borders. Other CN subnuclei could not be accessed without causing damage to the electrode array. Signals were amplified and digitized using TDT System 3 hardware. We quantified multi-unit activity (MUA) using a measure related to the voltage signal power in the frequency band occupied by extracellularly recorded action potentials. Specifically, multi-unit activity was measured by extracting the envelope of the band pass filtered voltage recordings as follows: 1) a band pass filter was applied between 300 Hz and 6000 Hz, 2) the absolute value was taken, 3) a low pass filter was applied below 300 Hz. A number of previous studies [23–27] have used similar or identical methods to derive an “analog measure of MUA” (aMUA) from the band-passed electrode signals. This approach is preferable to MUA measurements based on thresholding, as it does not require the choice of any free parameters and provides a substantially less noisy measure when compared to thresholding. The noisiness of the threshold MUA can be overcome by binning threshold crossings in relatively large time bins, but such binning precludes the study of neural activity with high temporal resolution.

For multi-unit recordings in the inferior colliculus (IC), a craniotomy was performed on the right side of the skull to expose the IC. The dura was removed and electrodes were inserted, oriented along the medial-lateral axis. Responses were recorded using 64-channel silicon electrode arrays (NeuroNexus) arranged in 8 shanks with 8 recording sites per shank. As with the DCN, we attempted to sample a large fraction of the IC across three dimensions in each animal by performing multiple electrode penetrations (typically 4 per animal and individually spaced at 100 μm intervals). Multi-unit activity was measured as described above.

Stimuli consisted of 50 ms tone pips with 2 ms rise and fall times presented once every 150 ms. Frequency response areas (FRAs) were estimated by varying the frequency of the tones from 2 to 90 kHz in 0.2 or 0.15 octave steps and the intensity in 5 dB steps from 15 to 100 dB SPL. In addition to tones, we recorded responses to 9 different representative CBA/Ca mouse vocalizations with typical durations between 30–60 ms presented at either 80 or 90 dB SPL [28]. The calls were chosen to be representative of the CBA/CaJ strain and other strains commonly used in biomedical research [4]. Stimuli were sampled at 192 kHz and delivered free-field through a speaker specially designed for the presentation of high ultrasonic frequencies (Vifa, Avisoft, Glienicke, Germany) positioned 10 cm from the ear contralateral to the recording site. A filter was applied to adjust the frequency response of the speaker such that it was flat (±10 dB) between 2 and 90 kHz when measured with an Avisoft CM16/CMPA condenser microphone placed where the animals head would be. No subharmonic distortions were detected for the communication calls or for tones at any of the frequency/level combinations tested in this study.

## Results

**Fig 1:**
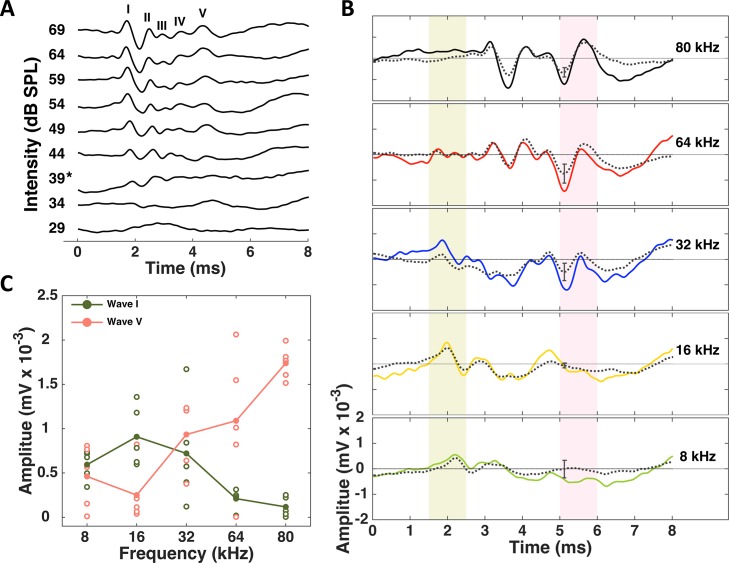
Auditory brainstem responses to click and tone stimuli. (A) ABRs recorded from one CBA/CaJ mouse in response to clicks of increasing intensity (individual traces from bottom to top). The star on the y-axis represents the threshold stimulus intensity. The different ABR peaks are labeled using roman numerals. (B) ABRs recorded from one CBA/CaJ mouse in response to pure-tone pips of increasing frequency (individual panels from bottom to top). Each panel shows the mean response (in color) averaged over 500 repeats. The black stippled line in each panel shows the mean response (± standard error) averaged over a population of 5 animals. The shaded areas in light green and pink indicate the timing of waves I and V respectively. (C) Amplitude of wave I vs. wave V at each of the frequencies we tested. Open circles represent the data obtained from individual animals. The lines join the mean values across different frequencies.

To study the frequency sensitivity of different areas along the ascending auditory pathway, we first recorded auditory brainstem responses to clicks (Fig 1A) and tones of varying frequencies (Fig 1B). ABRs are evoked potentials which typically occur within the first 10 ms after stimulus onset and consist of a series of positive-to-negative waves that reflect the synchronized neural activity in the auditory nerve and subsequent nuclei within the auditory brainstem [19, 29–31]. Fig 1B shows tone-evoked ABRs obtained from a representative mouse at 10 dB above threshold (solid lines) along with the ABRs averaged over all 5 animals in the sample population (black stippled lines). There were clear frequency-dependent differences in the ABR waveforms, to the extent that the early peaks were difficult to discern for high frequencies, and the late peaks were difficult to discern for low frequencies. To quantify the differences in the frequency sensitivity between peripheral and central areas, we measured the amplitude of wave I (green shaded area) and the amplitude of wave V (pink shaded area). We focused on waves I and V because they reflect the first stage of processing in the AN/CN and the final stage of brainstem processing in the IC, which serves as the hub of the central auditory pathway where inputs from all of the other brainstem nuclei are integrated for transmission to the thalamocortical circuit (for definition of waves I and V, see Methods). We found that across the population, increasing the stimulus frequency lead to a decrease in the amplitude of wave I. In contrast, the amplitude of wave V increased as the stimulus frequency was increased (Fig 1C; ANOVA, *p* < 0.01 for both waveform components). Tone-evoked ABRs can be difficult to interpret as they have not yet been widely studied in mice [18], but the increase in the amplitude of wave V relative to wave I with increasing frequency suggests an increase in IC activity relative to that in the periphery [32, 33]. A study of the likely cellular generators of ABR peaks (in cats) suggests that increasing how many neurons contribute and/or how strongly they synchronize their firing in response to sounds increases the amplitude of the corresponding ABR peak [34]. Thus, we hypothesized that the high ultrasonic frequencies that comprise many mouse vocalizations may be overrepresented in the central auditory system. We investigated this hypothesis in more detail using single- and multi-unit recordings to characterize the responses of neurons at different stages along the auditory pathway with high resolution.

**Fig 2:**
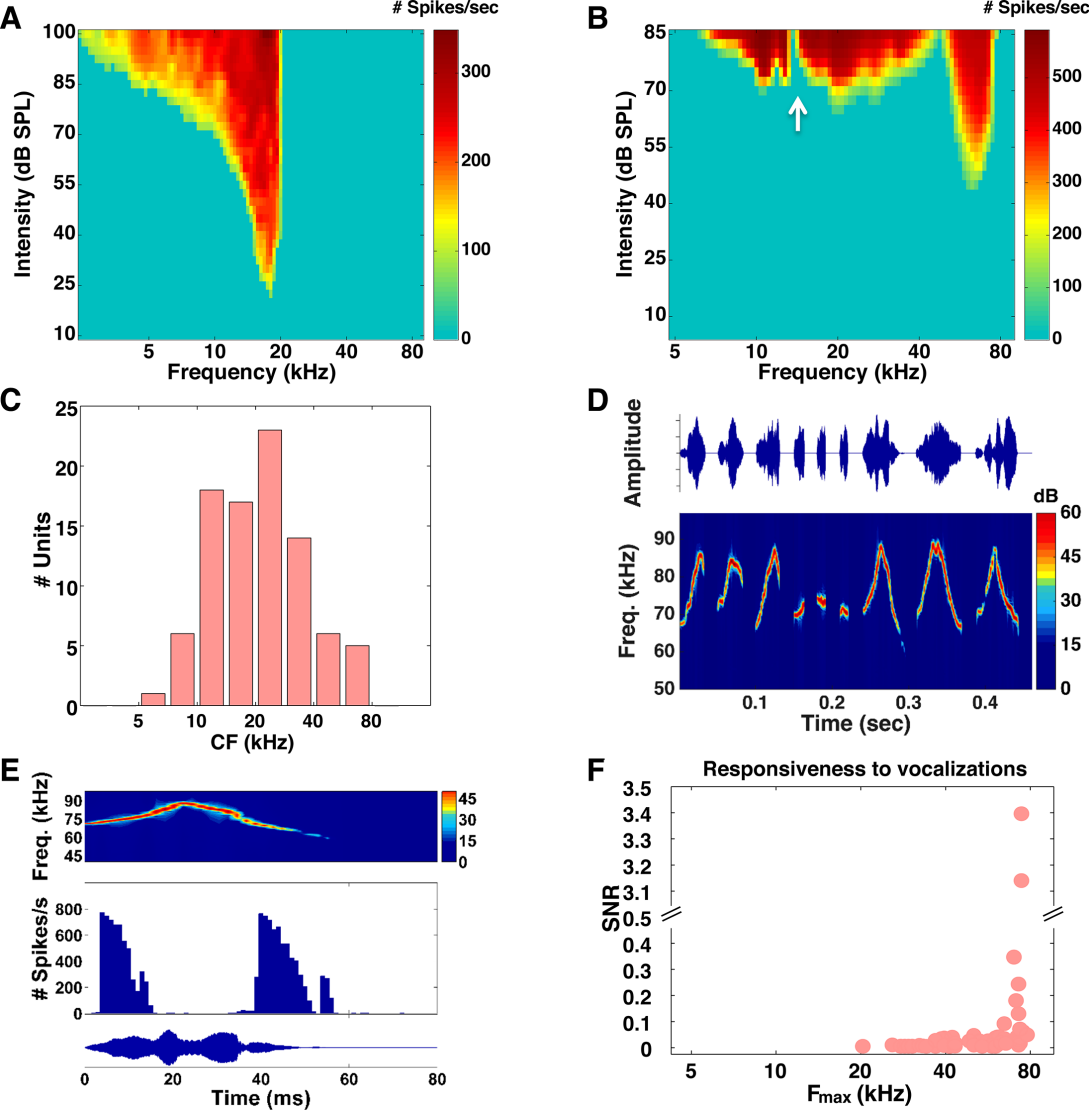
Auditory nerve responses to tones and vocalizations. (A) FRA of a representative auditory nerve fiber. FRAs were constructed from the responses elicited by tones of varying frequency (x-axis) and intensity (y-axis). (B) FRA of one AN fiber that was responsive to high frequency tones. The white arrow marks the location of a spectral notch created by the pinna. (C) Histogram of the distribution of CFs across the population of AN fibers. (D) Sound pressure waveforms (top panel) and spectrograms (lower panel) for the nine different CBA/Ca mouse vocalizations we tested. Note that all the vocalizations span a frequency range from 60–90 kHz. (E) PSTH of the response of the same fiber to a single mouse vocalization with a duration of 60 ms and averaged over 256 repeats. The spectrogram and sound pressure waveforms are shown at the top and bottom panels respectively. (F) Responsiveness to vocalizations. Each point indicates the SNR for one fiber as a function of the highest frequency for which its responses to tones were significantly greater than its spontaneous activity (F_max_). SNR values were averaged across the 9 different calls we tested.

We began by recording responses from the auditory nerve. We used established physiological criteria to differentiate AN fibers from CN neurons (see Methods and S1 Fig). We isolated 94 fibers recorded from 23 animals. Fig 2A and 2B show FRAs for two representative fibers (the spectral notches created by the pinna are evident in Fig 2B). Across our sample population, the most commonly occurring CFs were between 20 and 30 kHz (Fig 2C). This is consistent with the distribution of CFs observed in a previous study of the mouse AN [13]. We also recorded the responses of AN fibers to conspecific vocalizations with frequencies between 60 and 90 kHz (the sound pressure waveforms and spectrograms are shown in Fig 2D). To determine whether a vocalization evoked a significant response, we compared the distribution of driven spike counts to the distribution of spontaneous spike counts (Wilcoxon rank-sum test, *p* < 0.05). Fibers were regarded as responsive if statistically significant responses were evoked by at least 3 of the 9 different vocalizations we tested. Only a small number of fibers (7 out of 94 ≈ 7%; 95% CI via bootstrap resampling = [3% 12%]) responded to the vocalizations (the response of an example AN fiber is shown in Fig 2E). We quantified the responsiveness of each fiber to the vocalizations using a measurement of the signal-to-noise ratio (SNR) of the response, defined as the ratio of the variance of the PSTH to the average variance of the deviation from the PSTH on each trial. To determine the extent to which each fiber’s responsiveness to vocalizations correlated with its sensitivity to high ultrasonic frequencies, we determined the highest frequency for which its responses to tones were significantly greater than its spontaneous activity (F_max_). For the population of fibers we studied, only the small fraction with the highest F_max_ responded strongly to the vocalizations (Fig 2F).

**Fig 3:**
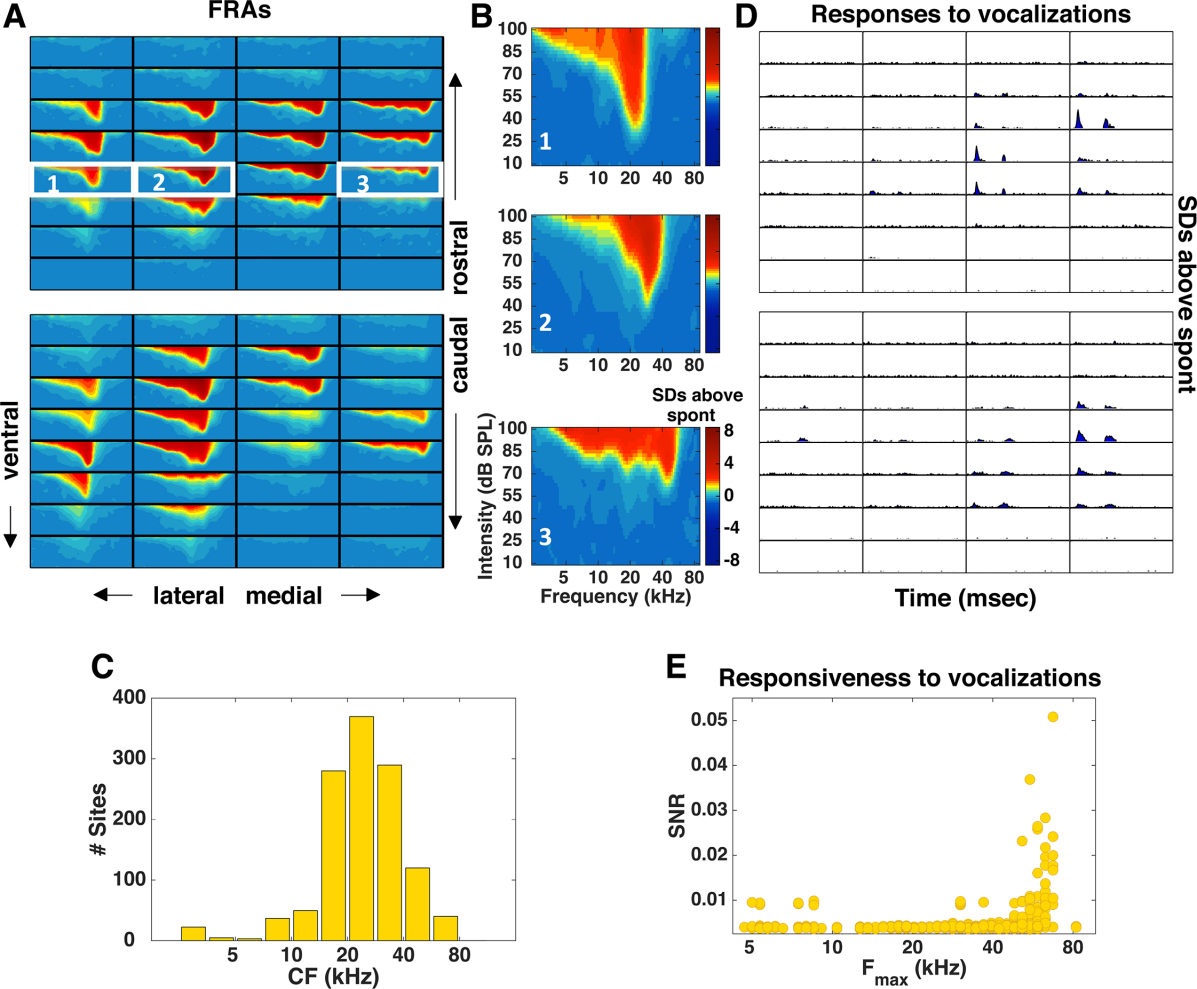
Multi-unit responses in the dorsal cochlear nucleus to tones and vocalizations. (A) Each panel shows FRAs for each of the 32 recording sites across one electrode penetration in the DCN. Two different penetrations are shown with the most rostral location at the top and the most caudal at the bottom. (B) FRAs estimated from multi-unit activity at three different recording sites along the medial-lateral axis in the DCN. The location of each site is indicated by the corresponding white rectangle in Fig 3A. (C) Histogram showing the distribution of CFs across the sites we recorded from in a population of 8 animals. (D) PSTHs of the responses to the vocalization stimuli for the two electrode penetrations for which FRAs were shown in (A). For each recording site, the x-axis (time) covers the range from 0 to 130 msec and the y-axis (SDs above spontaneous) covers the range from 0 to 5. (E) SNR for each recording site as a function of its F_max_. For each site, SNR values were averaged across the 9 different vocalizations tested.

We next investigated the representation of high ultrasonic frequencies in the dorsal cochlear nucleus by recording multi-unit activity using a large multi-electrode array. To sample the entire DCN, we performed multiple electrode penetrations oriented along the rostral-caudal axis (see Fig 3A for FRAs estimated from multi-unit activity for two example penetrations, and Fig 3B for FRAs estimated for three example sites). The distribution of CFs across all recordings sites in the DCN was centered around 20–30 kHz (Fig 3C), consistent with previous studies [35, 36]. As in the AN, only a small number of sites with sensitivity to high frequencies responded significantly to the vocalizations (118 recoding sites out of a total of 1248 ≈ 9%, 95% CI [5% 15%], see Fig 3D and 3E). The results from the AN and DCN are therefore consistent in suggesting that vocalizations evoke responses in only the small fraction of cells that are sensitive to high ultrasonic frequencies and, thus, are not well represented in the auditory periphery.

**Fig 4:**
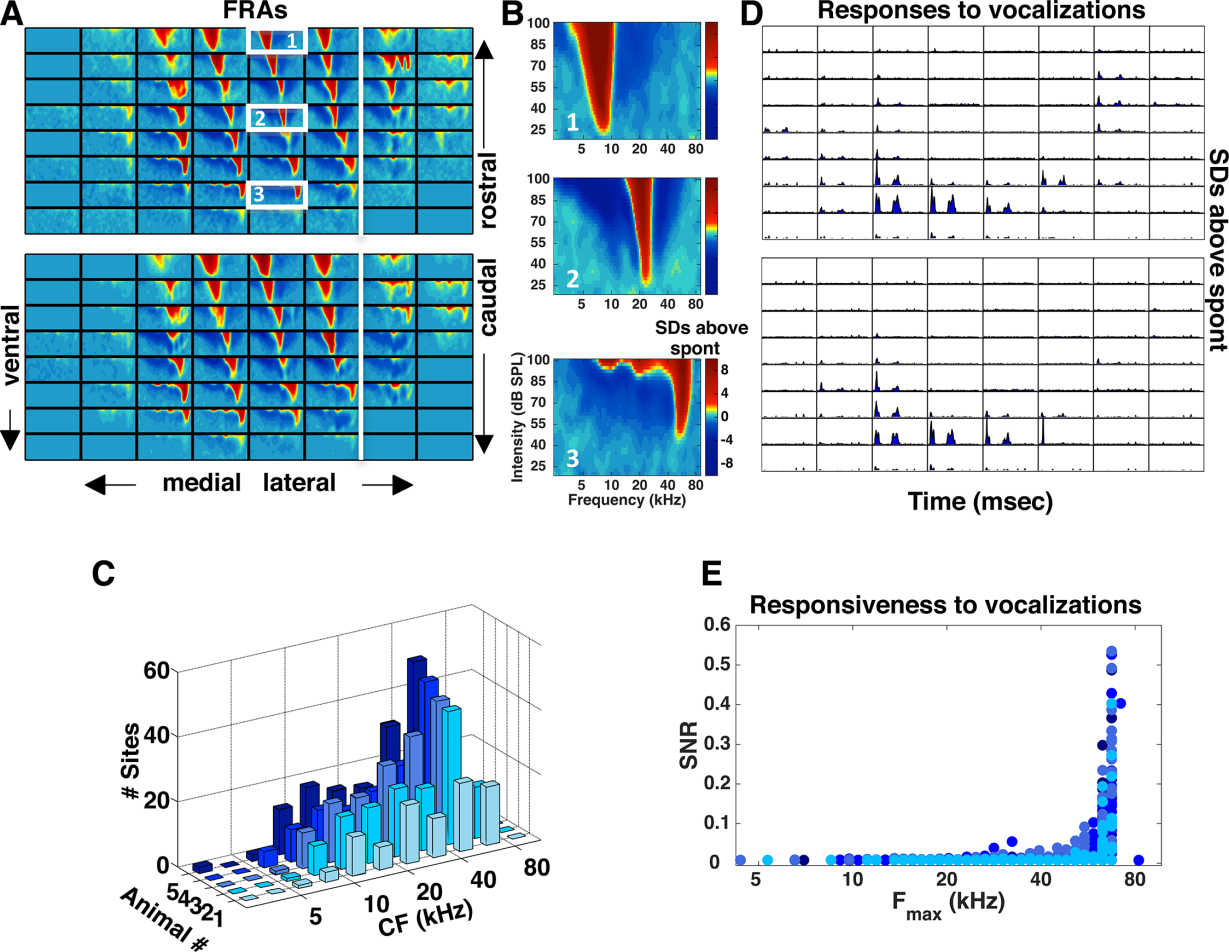
Multi-unit responses in the inferior colliculus to tones and vocalizations. (A) Each panel shows FRAs for each of the 64 recording sites across a single electrode penetration in the IC. Two different penetrations are shown with the most rostral location in the IC at the top and the most caudal location at the bottom. The white vertical line denotes the separation between the ICC and EIC. The borders between the ICC and the DIC were not easily distinguishable in most of the electrode penetrations we performed. (B) FRAs estimated from multi-unit activity at three different recording sites across a single penetration along the dorsal-ventral axis in the IC. Each location is indicated by the corresponding white rectangles in Fig 4A (top panel). (C) Histograms showing the distribution of CFs across the sites we recorded from the IC for each of 5 animals. (D) PSTHs of the responses to the vocalization stimuli for the two electrode penetrations for which the FRAs were shown in (A). For each recording site, the x-axis (time) covers the range from 0 to 130 msec and the y-axis (SDs above spontaneous) covers the range from 0 to 16. (E) SNR for each recording site as a function of its F_max_. For each site, SNR values were averaged across the 9 different vocalizations tested. Colors correspond to those in C.

Finally, we investigated the representation of high ultrasonic frequencies in the inferior colliculus of the midbrain. Like in the DCN, we performed multiple electrode penetrations in an attempt to sample the entire IC. The width of the probe (see Methods for further details) was sufficient to record responses from the different subnuclei, namely, the dorsal (DIC) and external (EIC) cortices, and the central nucleus (ICC). We distinguished the different subnuclei based on differences in the response properties between neighboring sites. For instance, the white vertical lines in Fig 4A separate the ICC from EIC, with the latter identified by its multi-modal FRAs (the border between the DIC and ICC was not clearly distinguishable in the penetrations shown in Fig 4A). We focused our analysis only on the ICC, which serves as the midbrain relay for the primary ascending auditory pathway. Unlike the AN and DCN, the most common CFs in the ICC were above 40 kHz. Fig 4C shows the distribution of CFs across all recording sites in the ICC for each of the five animals in our sample population. The CF distributions were not significantly different across animals (ANOVA, *p* > 0.7).

While only 7% and 9% of cells in the AN and CN exhibited statistically significant responses to vocalizations, we found that 59% of recording sites in the ICC (Fig 4D) responded to the communication calls (409 recording sites out of a total of 696, 95% CI [53% 66%]). Fig 4E plots the SNR as a function of F_max_ and further supports the finding that only sites with high F_max_ responded to the vocalizations. Thus, our results suggest that there is an overrepresentation of high ultrasonic frequencies in the ICC relative to the periphery, which renders this midbrain nucleus highly responsive to vocalizations.

## Discussion

We investigated the processing of high ultrasonic frequency communication calls in mice using ABR measurements in combination with single-unit and multi-unit recordings at three different stages along the auditory pathway. The two techniques are complementary, i.e., while the former provides, within the same animal, a coarse measure of the strength of the response across different relay nuclei, the higher resolution of the latter provides a better description of the response properties of neurons at each of these nuclei across different animals. The results obtained using these two approaches were consistent in suggesting the existence of a central overrepresentation of high frequencies that supports the processing of vocalizations, with high ultrasonic frequency tones and vocalizations exciting only a small fraction of neurons in the periphery, but a much larger fraction of neurons in the IC.

While our AN and CN results are consistent with previous studies of the mouse auditory periphery that have found few neurons sensitive to high ultrasonic frequencies [13, 35, 36], our finding that high ultrasonic frequencies are overrepresented in the IC differs from that of previous studies [11, 16]. For example, Stiebler and Ehret (1985) reported that the distribution of CFs in the IC was similar to that in the auditory nerve, with neurons with CFs above 45 kHz occupying only about 5–10% of the IC volume (see their Fig 4). In contrast, our results suggest that high ultrasonic frequencies are represented across a large fraction (59% of recording sites) of the medial and ventral portions of the ICC (see our Fig 4). The most likely explanation for the differences between our results and those of previous studies is the recording technology used; whereas previous studies relied on a single recording electrode, our multi-electrode array recordings enabled us to systematically sample the entire ICC across three dimensions in each animal.

An overrepresentation of behaviorally relevant frequencies has been observed in a number of other species, but the mouse seems to be unique in that this overrepresentation emerges centrally, rather than on the cochlea itself. The representation of behaviorally relevant frequencies is widely expanded on the basilar membrane of the horseshoe bat, *Rhinolophus ferrumequinu*m [37], the mustached bat, *Pteronotus parniellii* [38], and the African mole rat, *Cryptomys hottentotus* [39], and these overrepresentations are preserved in central structures, including auditory cortex [40–42]. It is unclear whether the overrepresentation of high ultrasonic frequencies in the mouse IC is preserved in primary auditory cortex; while there is a large region of auditory cortex dedicated to processing ultrasonic frequencies, there is some debate as to whether this region should also be considered ‘primary’ along with primary auditory cortex (A1) and the anterior auditory field (AAF) [43–45]. This issue, as well as the neural circuitry underlying the emergence of the overrepresentation in the IC, is a topic for future investigations.

Reports indicate that some auditory neurons are characterized by their ability to integrate information across different frequency bands and that these neurons are likely to contribute to the processing of complex sounds, particularly vocalizations [46]. These neurons, commonly referred to as combination-sensitive neurons, have been shown to exist in the midbrain of mice [47], bats [46, 48, 49] and birds [50, 51], and their nonlinear interactions can be either facilitatory or suppressive [46, 51]. In the mouse, it has been shown that almost 30% of the neural responses in the IC exhibit combination sensitivity with 16% reportedly being facilitatory and 12% inhibitory [47]. While these interactions are unlikely to play a role in the processing of the vocalizations we tested, which contained only a single frequency component, they may play an important role in coding other types of vocalizations or sounds.

## Supporting Information

S1 Fig Identification of auditory nerve fibers. Each point shows the relationship between the coefficient of variation (CV) of interspike intervals obtained from the responses to repeated presentations of tones at each fiber’s CF presented 30 dB above threshold, and the mode of the first spike latency (FSL) for every unit we recorded from. Different markers are used to group neurons according to the shapes of their PSTHs (see legend). The right and bottom panels show typical examples of primary, chopper, pri-N and onset PSTHs with their respective ISI histograms. Only cells with CVs ≥ 0.5, FSL ≤ 5 ms and primary-like PSTHs were regarded as AN fibers.
(TIF)
