# Supplementary figures and images for: An Overrepresentation of High Frequencies in the Mouse Inferior Colliculus Supports the Processing of Ultrasonic Vocalizations
Source: PLoS One. 2015 Aug 5;10(8):e0133251. doi: 10.1371/journal.pone.0133251 (PMC4526676; doi:10.1371/journal.pone.0133251)

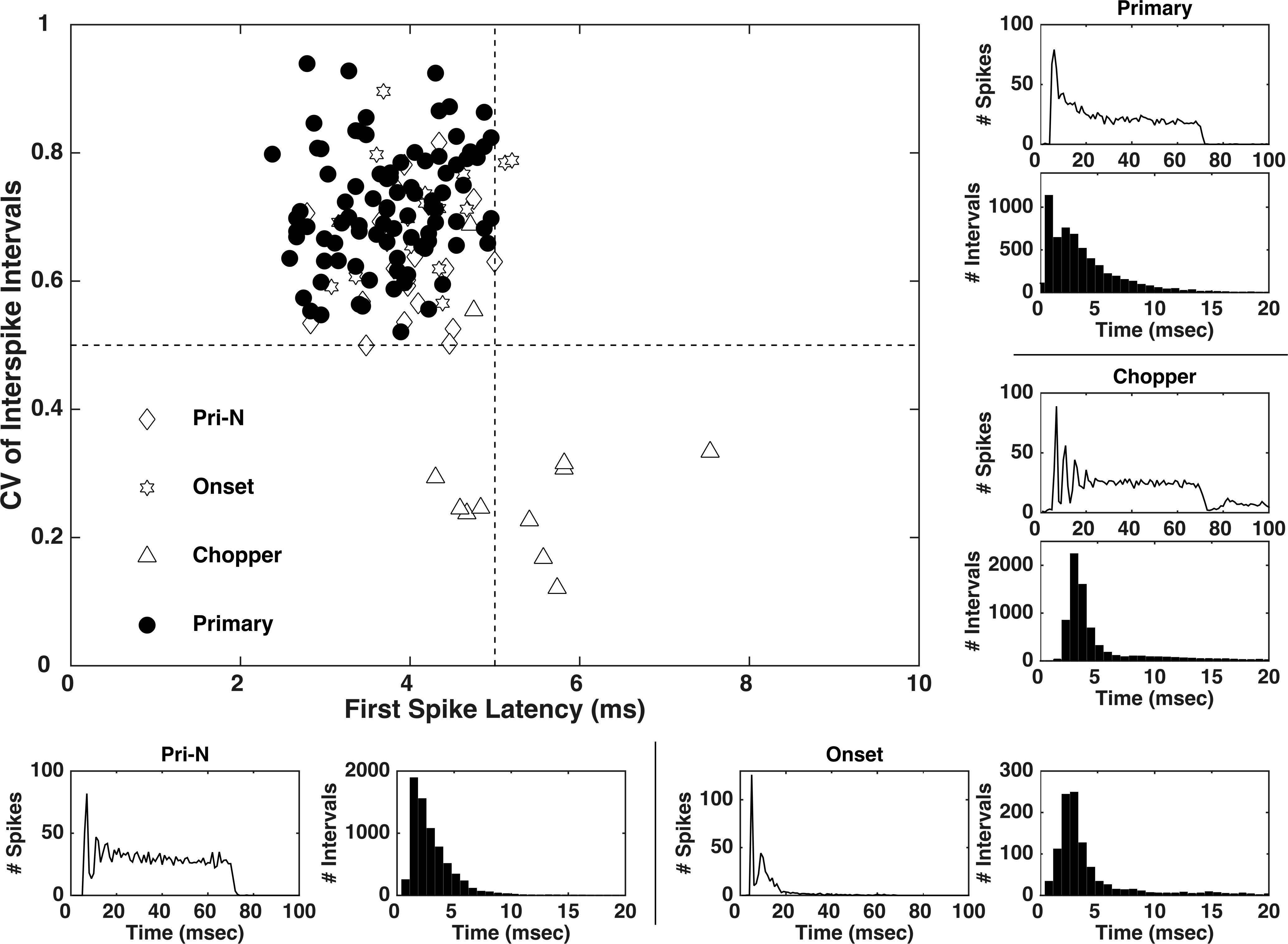

Supplement: S1 Fig — Each point shows the relationship between the coefficient of variation (CV) of interspike intervals obtained from the responses to repeated presentations of tones at each fiber’s CF presented 30 dB above threshold, and the mode of the first spike latency (FSL) for every unit we recorded from. Different markers are used to group neurons according to the shapes of their PSTHs (see legend). The right and bottom panels show typical examples of primary, chopper, pri-N and onset PSTHs with their respective ISI histograms. Only cells with CVs ≥ 0.5, FSL ≤ 5 ms and primary-like PSTHs were regarded as AN fibers. (TIF) [file pone.0133251.s001.tif]
